# A New Pedigree-Based SNP Haplotype Method for Genomic Polymorphism and Genetic Studies

**DOI:** 10.3390/cells8080835

**Published:** 2019-08-05

**Authors:** Zareen Vadva, Charles E. Larsen, Bennett E. Propp, Michael R. Trautwein, Dennis R. Alford, Chester A. Alper

**Affiliations:** 1 Program in Cellular and Molecular Medicine, Boston Children’s Hospital, Boston, MA 02115, USA; 2 Department of Pediatrics, Harvard Medical School, Boston, MA 02115, USA

**Keywords:** disease association, haplotype, HLA polymorphism, major histocompatibility complex (MHC), pedigree, phase, protocol, single nucleotide polymorphism (SNP), T1DGC, type 1 diabetes (T1D)

## Abstract

Single nucleotide polymorphisms (SNPs) are usually the most frequent genomic variants. Directly pedigree-phased multi-SNP haplotypes provide a more accurate view of polymorphic population genomic structure than individual SNPs. The former are, therefore, more useful in genetic correlation with subject phenotype. We describe a new pedigree-based methodology for generating non-ambiguous SNP haplotypes for genetic study. SNP data for haplotype analysis were extracted from a larger Type 1 Diabetes Genetics Consortium SNP dataset based on minor allele frequency variation and redundancy, coverage rate (the frequency of phased haplotypes in which each SNP is defined) and genomic location. Redundant SNPs were eliminated, overall haplotype polymorphism was optimized and the number of undefined haplotypes was minimized. These edited SNP haplotypes from a region containing *HLA-DRB1* (DR) and *HLA-DQB1* (DQ) both correlated well with HLA-typed DR,DQ haplotypes and differentiated HLA-DR,DQ fragments shared by three pairs of previously identified megabase-length conserved extended haplotypes. In a pedigree-based genetic association assay for type 1 diabetes, edited SNP haplotypes and HLA-typed HLA-DR,DQ haplotypes from the same families generated essentially identical qualitative and quantitative results. Therefore, this edited SNP haplotype method is useful for both genomic polymorphic architecture and genetic association evaluation using SNP markers with diverse minor allele frequencies.

## 1. Introduction

Evidence that specific markers in or near candidate susceptibility genes mark susceptibility to type 1 diabetes (T1D) was first obtained by association studies, wherein positivity rates of major histocompatibility complex (MHC) alleles in patients were compared with those in an “ethnically-matched” control population (so-called standard “patient vs. control” association studies) [1,2]. Variations on such patient vs. control association studies are still widely favored [3,4] for studying this complex genetic disease [5,6,7]. However, results from patient vs. control association studies can be confounded by population stratification [8,9,10]. Ethnic matching of patients and control subjects helps to reduce confusion of a purely subpopulation genetic marker (that could be increased in populations at elevated risk for disease), with a genetic marker for a susceptibility gene, that is often also a subpopulation marker when disease incidence differs considerably among ethnic subpopulations.

Thirty-five years ago, we developed a method that minimizes genetic association study population stratification using a family-based haplotyping approach to determine the frequencies of alleles and haplotypes in T1D-affected pedigrees [11]. The “disease vs. family control haplotype” method yielded sets of T1D (DIS; occurring in patients) and family control (FC; not found in any patient in the family) haplotypes for comparison. The underlying haplotyping method was originally implemented using the HLA and MHC complement gene (“complotype”) typing to identify megabase (Mb)-length haplotypes fixed (i.e., at relatively high frequency) in a population (i.e., ancestral (AHs) or conserved extended haplotypes (CEHs)) and their regional haplotypic fragments [12,13,14]. The population-level existence of AHs/CEHs and their regional MHC fragments has been validated repeatedly using pedigree-based haplotyping methods, but CEHs are often undetectable using maximum likelihood techniques based on underlying data from unrelated subjects [15,16].

Here, we adapted that pedigree-based method to create a modern version based only on single nucleotide polymorphism (SNP) data. A validated method of this type should be useful in future studies both within and outside the human MHC to study both short- and long-range population-level haplotype sequence fixity and as a source for genetic association assays. We chose to validate the method using MHC data because: (a) of the availability of overlapping HLA and SNP typing data from two earlier studies; and, (b) of the vast prior information available from this region including its significant population-level genetic polymorphism and the long-range haplotype sequence fixity in many populations (including among families with members that are affected by T1D).

We used both the Type 1 Diabetes Genetics Consortium (T1DGC) MHC Fine Mapping study (containing biallelic dense SNP [17,18] and polymorphic HLA allele [19,20] genotypes) and the T1DGC ImmunoChip study (containing primarily biallelic dense SNP genotypes [21]) databases. Both databases provided data collected from T1D-affected subjects, their siblings and their parents. A subset of pedigrees overlapped in the two databases. We used the ImmunoChip study database to generate pedigree-phased dense SNP haplotypes that were assigned DIS or FC status by the original methodology within a 240 kb region showing the strongest genetic association to T1D within the human genome [3,4,22] containing the genes *HLA-DRB1* (DR), *HLA-DQA1* and *HLA-DQB1* (DQ) (together, the HLA-DR/DQ region). We optimized the selection of SNP data for haplotype analysis from a larger SNP dataset based on minor allele frequency (MAF) variation and redundancy, coverage rate and genomic location. Finally, we compared those edited SNP haplotype variants with pedigree-analyzed classically-typed HLA-DR,DQ haplotypes from the same families that were available from the earlier MHC Fine Mapping study, in order to test their relative ability to detect genetic association with T1D.

## 2. Materials and Methods

Our goal was to design a method to convert SNP genotype data obtained in families (pedigrees) into phased haplotypes edited to remove redundant and less informative SNPs to produce an optimized final set of unambiguous fully pedigree-phased edited SNP haplotypes useful for a variety of genetic and genomic assays. The new core method of this process (Section 2.3) is based on optimization, namely which SNPs to remove (“triage”) and which to maintain in the finalized edited haplotypes. We describe a step-wise process for the creation of these edited SNP haplotypes. We then present an alternative method. As a direct test of the efficacy of the method, we test the extent to which the edited 27-SNP haplotypes correlated with the specific classically-defined 4-digit HLA pedigree-phased haplotypes. The final section describes one application of these haplotypes: a previously described family-based genetic association assay for T1D, using either edited SNP or classically-defined *HLA*-*DRB1*, -*DQA1*, -*DQB1* haplotypes for the same region of the MHC.

### 2.1. T1DGC Datasets

Two different T1DGC datasets were analyzed in this study. Both studies contain mostly families with multiple-affected children from several geographical cohorts. The MHC Fine Mapping dataset (June, 2009 (final) data freeze) consisted of 2298 families from nine geographical cohorts: Asia-Pacific (AP), British Diabetic Association, Danish, Europe (EUR), Human Biological Data Interchange, Joslin, North America (NA), United Kingdom (UK) and Sardinia. The MHC Fine Mapping study provided both 4-digit HLA and SNP genotyping data. The T1DGC ImmunoChip dataset (dbGaP Study Accession: phs000911.v1.p1) consisted of 2708 families from four of the same geographical cohorts: AP, EUR, NA and UK, and it provided dense SNP typing data alone. Only 2609 of those families were affected sib pair families having at least two children with T1D. The dataset also included 19 families with only one affected child and 35 families with no T1D-affected member. A total of 1067 families were shared between both T1DGC datasets.

### 2.2. Genotype Extraction and Pedigree-Phased Haplotypes

**Figure 1:**
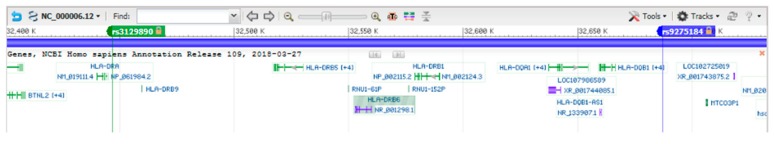
Genomic map of HLA-DR/DQ region in the human major histocompatibility complex (MHC) reference sequence. The map shows a slightly larger region than that phased in MERLIN. The two marked single nucleotide polymorphisms (SNPs) represent the boundaries of the phased 101 SNP haplotypes from which SNPs were “pre-triaged” for redundancy to create the initial 37-SNP haplotypes for further editing.

PLINK [23] extracted and combined family demographic, phenotypic and genotypic data from the T1DGC ImmunoChip database in a genomic region stretching from *HLA-DRA* to *MTCO3P1* (Figure 1) to create a standard pedigree file. Unless stated otherwise, all SNP position (pos) data are for human chromosome 6 from dbSNP build GRCh38.p12. The boundary SNPs were rs14004 (pos: 32439932) and rs3104402 (pos: 32713899). Thus, the region length was nearly 274 kb. PLINK determined the total genotyping rate to be 0.998885, with all 217 SNPs and 10791 subjects passing filters and quality-control measures. Separate analyses of the same final region, but using data in which the region extracted in PLINK and later phased was significantly larger, gave essentially identical results in downstream studies (data not shown).

Family genotype data and 1383 non-genotyped (missing) founder placeholders were phased from the pedigree file into haplotypes using MERLIN (version 1.1.2) [24]. We used the “best” haplotype estimation mode in MERLIN to provide us with haplotypes that correspond to the most likely pattern of gene flow. We then analyzed the phased haplotypes of a sub-region containing 101 contiguous SNPs in the HLA-DR/DQ region. The SNPs ranged from rs3129890 to rs9275184 (Figure 1). Haplotype crossovers for each family were assessed by determining instances in which a haplotype changed from the first to last SNP in the 101 SNP HLA-DR/DQ region, and families in which crossovers occurred were subsequently removed from further analysis. We removed the few families with such apparent crossovers for two reasons: (a) apparent de novo haplotype crossovers (i.e., within the families studied) occasionally are inaccurate and can occur due to de novo mutations or rare SNP typing or MERLIN phasing errors, and we wished to minimize such complexities; and, (b) the method described here is not intended to identify de novo haplotype crossovers. Although our method is directed at identifying population-level common and rare SNP haplotypes, the output of the method would be useful for comparison with output from those rare families with apparent crossovers for detecting and/or validating de novo haplotype mutations or crossovers. Finally, we note that genotyping errors (considered extremely infrequent in these two datasets) would likely have only minor effects on the results for the common and minor variants we studied (as genotyping errors would result in either unphased or singleton variants).

### 2.3. Creating Finalized Founder SNP Haplotypes in the HLA-DR/DQ Region

Using the phased founder (i.e., parental) SNP haplotypes in our dataset, we designed a work flow to remove (“pre-triage”) SNPs to increase the number of fully-phased haplotypes. A “fully-phased” haplotype is a haplotype defined at every SNP (i.e., assigned a phased nucleotide at every SNP position). Phased coverage at every SNP was first quantified for the entire set of founder haplotypes. “Coverage” is the percentage of all haplotypes that contain an assigned (i.e., phased) nucleotide at any given SNP. Separately, SNP MAF was provided by T1DGC for every SNP (all of which were biallelic). Six SNP MAF categories were used to create separate SNP groups for pre-triage. We arbitrarily decided to set a preliminary goal of retaining only 36–37% of all the SNPs within the region to optimize resultant haplotype diversity, coverage and SNP spatial distribution. We chose a bell-shaped distribution of MAFs for the initial pre-triage such that a higher percentage of the final SNPs would be in the three categories between 11% and 40% MAFs and fewer in the 1–10% and 41–50% categories. Within each MAF category, SNPs with higher coverage rates were retained unless the resulting spatial distribution within the region would be grossly asymmetric. Thus, priority was given to higher coverage. Supplementary Table S1 shows the 37 SNPs chosen for the original analysis and the 10 SNPs edited out in the following step. The MAF distribution of these SNPs was four in the 1–5% range, three in the 6–10% range, six in the 11–20% range, 12 in the 21–30% range, seven in the 31–40% range and five in the 41–50% range.

After the pre-triage step, we sorted haplotype sequences to isolate the fully-phased haplotypes. The overall coverage rate for all SNPs ranged from 79.1 to 90.9%. We then sorted the fully-phased SNP haplotype variants from highest to lowest frequency and tested for SNP redundancy among the haplotype variants. We then determined, for each SNP in a given MAF range, the haplotype at which it had a different allele from the first haplotype. If there was a SNP that changed alone in any variant among the group of haplotypes comprising the top 90%, then it was kept. For SNP allele pairs (or higher groupings) that changed together among the SNP haplotypes, we determined whether the SNPs were biallelic as a unit (i.e., whether they existed as only two variants among all haplotypes). If SNP pairs (or larger groupings) were biallelic among the top 95% of all haplotypes (i.e., were “redundant”), then the SNP(s) with the lower coverage was/were eliminated. Re-sorting founder haplotypes based on each new set of SNPs, sorting the haplotypes by highest to lowest frequency, and checking for additional SNP redundancy was repeated until the SNP redundancy was eliminated.

### 2.4. An Alternative Triaging Method

We tested an alternative SNP-editing haplotype method in which there was no pre-triaging of SNPs to determine whether the numbers and polymorphic complexity of the resultant edited haplotypes differed significantly. Thus, the method began in the last paragraph of Section 2.3 beginning with all 101 SNPs from the HLA-DR,DQ region (instead of only the 37 shown in Supplementary Table S1), and the triaging process in the last step resulted in a final number of 39 SNPs in edited haplotypes (data not shown). Several parallel studies were conducted with these haplotypes for comparison with our main 27-SNP edited haplotype results, and the downstream results were similar.

### 2.5. Identifying SNP Haplotype Variants for MHC CEHs and Identifying CEHs from SNP Haplotype Variants Based on the T1DGC MHC Fine Mapping Study

HLA (at the four-digit level) and SNP genotyping data from the MHC Fine Mapping study were provided by T1DGC. As described previously [25], the T1DGC HLA typing methodology did not target all polymorphic sites. Some alleles were not distinguished. For example [25], *HLA*-DQB1*02:01, found on DR3 haplotypes, and *HLA*-DQB1*02:02, found on DR7 haplotypes, were both assigned the *02:01 allele in the T1DGC data. Here, we maintain that assignment when referring to T1DGC data, but we provide the appropriate alleles [13,14,26] in named CEHs or their HLA-DR,DQ fragments. All genotyping data for the MHC region were phased together in MERLIN.

Two CEHs ([HLA-B8,SC01,DR3] and [HLA-B18,F1C30,DR3]) are at particularly high frequency among European Caucasian families affected by T1D, and we had prior knowledge that these two CEHs differed in or near the HLA-DR/DQ region [14,27]. To enhance our ability to differentiate the HLA-DR,DQ variants of these two CEHs in haplotypes lacking or unphased for either of the two HLA-C,B fragment variants distinguishing them, we analyzed 524 B8,DR3 and 214 B18,DR3 haplotypes fully defined at *HLA*-*C*, -*B*, -*DRB1*, -*DQA1*, and -*DQB1* to identify five SNPs in the MHC Fine Mapping study useful as SNP haplotype surrogates (Table 1). These SNPs are located both telomeric to and within the genomic region used from the T1DGC ImmunoChip data in the main results presented here. Although each of the two CEHs were represented by some minor 5-SNP haplotype variants (Table 1), none of the B8,DR3 haplotypes had the dominant B18,DR3 5-SNP haplotype and none of the B18, DR3 haplotypes had the dominant B8,DR3 5-SNP haplotype.

In several other cases, we performed a reverse analysis using the MHC Fine Mapping data. When a specific HLA-DR,DQ haplotype had a relatively high-frequency 27-SNP haplotype identified from the T1DGC ImmunoChip data, we analyzed the *HLA*-*C* and *HLA*-*B* alleles of both the dominant and most frequent minor 27-SNP haplotypes using the HLA typing data from the MHC Fine Mapping study for the 1067 families overlapping between the studies.

### 2.6. Correlating Edited SNP Haplotypes and HLA Haplotypes Overlapping in the Two T1DGC Datasets

We used two methods to correlate the dominant HLA-DR,DQ haplotypes determined in the MHC Fine Mapping study with the major edited 27-SNP haplotypes determined from the ImmunoChip dataset using the 1067 families shared between the two T1DGC datasets. We determined first the dominant HLA-DR,DQ haplotype corresponding to each major edited 27-SNP haplotype. Separately, we quantified the percentages of the two most frequent edited 27-SNP haplotypes along with the percentage of unphased (at even a single SNP) 27-SNP haplotypes corresponding to each of the major classically-defined HLA-DR,DQ haplotypes.

Finally, we compared the 27-SNP haplotypes and the HLA-typed DR,DQ haplotypes in these shared families for statistical results in the T1D gene association assay both in terms of ranking of and relative numbers of haplotypes distributed between DIS and FC designations. To perform the gene association assay based on HLA-DR,DQ haplotypes in the MHC Fine Mapping study, we categorized the haplotypes based on their 4-digit alleles and then combined them into haplotype groups based on a nomenclature presented previously [3]. We categorized only those haplotypes (>97% of all haplotypes) that correlated with the major edited 27-SNP haplotypes determined from the ImmunoChip dataset (Section 2.6). We calculated a DIS/FC haplotype ratio of the HLA-DR,DQ haplotypes and compared it with the *HLA*-*DRB1*-*DQB1* patient/control (P/C) ratio for T1D susceptibility presented previously [3]. Both ratios were also compared based on the relative rank of the haplotypes. We defined a haplotype with a DIS/FC ratio >1 as a susceptibility haplotype and a haplotype with a DIS/FC ratio <0.5 as a protective haplotype, with neutral haplotypes falling within a DIS/FC ratio between 0.5 and 1.0.

### 2.7. Assigning Disease and Family Control Status to Haplotypes for a Genetic Association Assay

Using the final set of all fully-phased edited 27-SNP haplotypes, we assigned DIS and FC status to founder haplotypes. A DIS haplotype was defined as any parental haplotype in a patient with T1D. A FC haplotype was defined as any parental haplotype only in unaffected members of the same family. Subjects assigned unknown disease status were treated as unaffected members of the pedigree. Finally, to equalize the number of DIS and FC haplotypes based on their parental contribution, we removed any founder lacking either a DIS or FC haplotype. Thus, only haplotypes from founders who had one DIS and one FC haplotype were retained. This was designed to maximize ethnic identity distribution between DIS and FC haplotypes.

### 2.8. Statistical Analyses

DIS and FC SNP haplotypes were ranked separately based on their frequencies within each of the two categories. Pearson’s chi-squared (χ^2^) test was performed to determine whether there was a statistical difference between the raw number (*n*) distribution of identical DIS vs. FC SNP haplotypes if DIS and FC haplotypes were each observed at *n* ≥ 5. Significance was set at *p* < 0.05. A Bonferroni correction was applied to adjust for significance for multiple comparison tests.

## 3. Results

### 3.1. Identifying Fully-Defined Edited SNP Haplotypes from the T1DGC ImmunoChip Study

The MERLIN output for the T1DGC ImmunoChip dataset was 10790 founder haplotypes containing 101 SNPs in the region (Figure 1). Of those haplotypes, 913 were undefined at all positions and many haplotypes were either partially undefined or unphaseable due to missing pedigree genotype data. Due to MERLIN-assigned haplotype crossovers within the studied region, 114 families (4.2% of all families in the dataset) were removed from further analysis.

The pre-triage method used to select SNPs resulted in 6194 fully-defined (at every SNP) 37-SNP haplotypes (57% of the original haplotypes). Upon further removal of 10 redundant SNPs (Supplementary Table S1), the number of fully-defined haplotypes increased to 6309 27-SNP haplotypes (58% of the original haplotypes). Among the 6309 haplotypes were 94 unique haplotype variants. Of these, 15 variants each existed above 1% (Table 2) and 41 variants were single examples (<1% of all haplotypes).

As compared with the pre-triage results, the non-pre-triage method resulted in fewer fully-defined 39-SNP haplotypes (*n* = 5695). Most of the results presented in the rest of this report, therefore, focus on the fully-defined 27-SNP haplotypes resulting from the pre-triage method.

### 3.2. Comparison of Overlapping Families in T1DGC Studies: Testing SNP Haplotype Method vs. HLA Typing

Of the 6309 27-SNP haplotypes from the entire T1DGC ImmunoChip dataset (Table 2), 2561 (41%) were from families also in the T1DGC MHC Fine Mapping database. Of those 27-SNP haplotypes shared by the two studies, 2466 (96.3%) were among the top 19 variants: Table 3 shows the total numbers of 27-SNP haplotypes for each of those major variants along with the 4-digit alleles or 2-digit specificities of the major HLA-DR,DQ haplotypes, groups or fragments that dominated them. Each variant was dominated by a particular HLA-DR,DQ haplotype or haplotype group. For example, the most common variant among the group, variant 1, was the HLA-DR4,DQ8 haplotype (a group specificity composed of haplotypes containing a wide variety of DR4 alleles (e.g., *HLA*-DRB1*04:01, *04:02, *04:03) in addition to *HLA*-DQA1*03:01 and *HLA*-DQB1*03:02). In contrast, the second most common variant, variant 2, was predominantly *HLA*-DRB1*03:01, -DQB1*02:02 (DR3,DQ2) fragments of the [HLA-B8,SC01,DR3] CEH, but the DR3,DQ2 fragment of the [HLA-B18,F1C30,DR3] CEH was variant 4. Two DR,DQ haplotypes (*HLA*-DRB1*13:02, -DQB1*06:04 and *HLA*-DRB1*13:01, -DQB1*06:03) each dominated two other 27-SNP haplotype variant groups (variants 10 and 19 and variants 11 and 13, respectively).

Table 4 and Supplementary Table S3 show the opposite information to that of Table 3: the degree to which a particular dominant 27-SNP haplotype from Table 3 represented the entire group of HLA-DR,DQ haplotypes (as defined by fully-phased *HLA-DRB1*, *-DQA1*, *-DQB1* alleles) was remarkably high. Except for the three DR,DQ haplotypes mentioned above that were found in two different dominant 27-SNP haplotypes, few to none of the most frequent HLA-DR,DQ haplotypes contained a secondary 27-SNP haplotype variant (Table S3). Most of the differences between the total numbers of specific HLA-DR,DQ haplotypes and the total numbers of the dominant 27-SNP haplotype representing those DR,DQ haplotypes were caused by the failure of full phasing among the 27 SNPs (Table S3). Thus, the major 27-SNP haplotypes correlated directly with the major HLA-DR,DQ haplotypes.

### 3.3. Summary of Edited SNP Haplotypes Distinguishing DR,DQ Haplotypes and Specific CEHs that Share HLA-DR,DQ Alleles

Table 5 shows, by direct comparison, the high degree to which the major 27-SNP variants directly correlated with specific HLA-DR,DQ haplotypes or haplotypic groups. For 15 of the 19 most common 27-SNP variants, 95% or more of all individual haplotypes in the group were part of a single HLA-DR,DQ haplotype and four (variants 1, 6, 8 and 18) comprised a haplotypic group, and all 19 of the top 27-SNP variants reach the 85% or higher level of this metric.

As with HLA-DR,DQ 4-digit allelic haplotypes, there is a dominant long-range CEH specific for most 27-SNP haplotype variants (Table 5). Furthermore, two major 27-SNP variants distinguish different CEH fragments of three HLA-DR,DQ haplotypes: HLA-DR3,DQ2 by SNP variants 2 and 4; HLA-DR1302,DQ0604 by SNP variants 10 and 19; and HLA-DR1301,DQ0603 by SNP variants 11 and 13. The CEHs represented by variants 2 and 4 are well known, and variant 10 is well characterized [13,14]: the class I fragment alleles are (*HLA*-C*03:04,B*40:01) and its complotype is SC02. The putative CEH represented by variant 19 (Table 5) has not been previously characterized. SNP variant 19’s class I fragment alleles are (*HLA*-C*07:01,B*15:17)—a rare centromeric class I haplotype. The CEHs represented by variants 11 and 13 are also less well characterized. The variant 11 CEH ([HLA-C12,B38,SC21,DR1301,DQ0603]) is a class II variant of the well-known Ashkenazi CEH [HLA-C12,B38,SC21,DR0402,DQ0302] (unpublished observations), but they appear to differ elsewhere in class I as well: the DR4,DQ8 CEH is dominated by *HLA*-A*26:01 [13,14], whereas six of the ten variant 11 DR13, DQ6 CEH examples (Table 5) bear *HLA*-A*02:01 (two others bear *HLA*-A*26:01). The variant 13 putative CEH ([HLA-C0303,B1501,unk,DR1301,DQ0603] may be a class II variant of either of two previously identified DR4 AHs [13].

Thus, 27-SNP haplotype variants may be useful in identifying previously unidentified or only partially characterized AHs/CEHs. Other than the ones mentioned above, another putative CEH [HLA-C7,B39,unk,DR8], represented by variant 9, has not been, to our knowledge, previously characterized. Of the 11 examples we found of this haplotype group, nine had the (*HLA*-C*07:02,B*39:06) fragment (six with *HLA*-A*24:02) and the other two contained the class I haplotype (*HLA*-A*02:01,C*07:02,B*39:01). As another example, the putative CEH [HLA-C12,B39,unk,DR16] of variant 12 has also not been described previously. Of the nine examples of this putative CEH, seven had the (*HLA*-C*12:03,B*39:01) centromeric class I fragment and the other two contained the class I haplotype (*HLA*-A*02:01,C*12:03,B*39:06).

Some edited 27-SNP haplotypes contain a secondary CEH or putative CEH. For example, the variant 7 SNP haplotype has a secondary well-characterized CEH ([HLA-C12,B18,S042,DR15,DQ6]; *n* = 8 (13% of 60 total DR15, DQ6 haplotypes evaluated)). A second variant 12 haplotype (*n* = 7; 18% of all variant 12’s defined HLA-DR,DQ haplotypes) may be a CEH: [HLA-C7,B44,unk,DR16] with the centromeric class I fragment (*HLA*-C*07:04,B*44:02). Finally, three other previously unreported putative CEHs contain, at 4-digit resolution, the following class I fragments: (a) SNP variant 15: (*HLA*-C*07:02,B*07:02) (although this is a common Caucasian HLA-C,B fragment); (b) SNP variant 17: (*HLA*-C*05:01,B*44:02); and, (c); SNP variant 18: (*HLA*-C*04:01,B*35:01). Further work (e.g., sequence analysis in class III) is required in order to confirm the CEH status for each of these apparently fixed long-range haplotypes.

### 3.4. Establishing and Analyzing the Designated DIS and FC SNP Haplotypes in the T1DGC ImmunoChip Study and Analyzing SNP Haplotypes for Genetic Association with T1D

Of the 6309 fully-defined 27-SNP haplotypes, 4272 were DIS and 2037 were FC haplotypes, comprised of 94 different haplotype variants (*n* = 62 DIS and *n* = 69 FC variants). We removed 603 founders who had no FC haplotype. With 27-SNPs, 5364 fully-phased SNP-haplotypes (*n* = 3360 DIS and 2004 FC haplotypes) remained. There were 87 different haplotype variants (*n* = 61 DIS and *n* = 61 FC variants), including 45 singleton haplotypes (<1% of all haplotypes). The most common DIS haplotype was variant 1 (*n* = 1244, 37% of all DIS haplotypes), and the most common FC haplotype was variant 7 (*n* = 254, 13% of all FC haplotypes).

We then equalized the number of DIS and FC haplotypes, using only founders with one DIS and one FC haplotype, which resulted in 2004 DIS and 2004 FC haplotypes. There were 75 different haplotype variants (*n* = 43 DIS and *n* = 61 FC variants). The most common DIS haplotype was variant 1 (*n* = 916, 46% of all DIS haplotypes), and the most common FC haplotype remained as variant 7 (Table 6). Among the haplotypes shown in Table 6 (where *n* ≥ 5), Pearson’s chi-squared test showed a statistically significant difference between DIS and FC SNP haplotype frequencies (*χ*^2^ = 1198.15, df = 14, *p* = 4.34 × 10^−247^; *p*-adjusted = 6.51 × 10^−246^).

#### 3.4.1. Analyzing Genetic Association with T1D among Families Overlapping in the Two T1DGC Studies Using the Designated DIS and FC SNP Haplotypes from the ImmunoChip Study

Using only overlapping families from both the MHC Fine Mapping and ImmunoChip datasets, we observed 2561 fully-phased 27-SNP haplotypes, including 1747 DIS and 814 FC haplotypes. We then equalized the number of DIS and FC haplotypes, keeping haplotypes based on parental contribution to the patients, which resulted in 808 DIS and 808 FC haplotypes (Table 7). This group of 27-SNP haplotypes was composed of 48 different variants (*n* = 26 DIS and *n* = 42 FC variants). The most common DIS variant was variant 1 (*n* = 373, 46% of all DIS haplotypes), and the most common FC variant was variant 7 (*n* = 103, 13% of all FC haplotypes).

Pearson’s chi-squared test showed a statistically significant difference between DIS and FC SNP haplotype frequencies (*χ*^2^ = 330.04, df = 9, *p* = 1.09 × 10^−65^; *p*-adjusted = 1.09 × 10^−64^). The results of these tests performed in overlapping families between the two T1DGC datasets largely mirror the results of the SNP haplotype method and genetic association assay performed on the entire ImmunoChip dataset (see Section 3.4).

#### 3.4.2. Analyzing Genetic Association with T1D among Families Overlapping in the Two T1DGC Studies Using the Designated DIS and FC HLA-DR,DQ Haplotypes from the MHC Fine Mapping Study

To compare the statistical results of our genetic association assay based on the edited 27-SNP haplotypes in the overlapping families from both T1DGC datasets, we performed a genetic association analysis using the same families using only the HLA-DR,DQ typing to determine the haplotype identities from the MHC Fine Mapping dataset. We initially identified 3735 HLA-DR,DQ haplotypes (*n* = 2564 DIS and *n* = 1171 FC haplotypes). We then equalized the number of DIS and FC haplotypes, keeping haplotypes based on parental contribution to the patients, which resulted in 1171 DIS and 1171 FC haplotypes (Table 8). The most common DIS haplotype group was DR4,DQ8 (*n* = 508, 43% of all DIS haplotypes), and the most common FC haplotype was DR15,DQ0602 (*n* = 166, 14% of all FC haplotypes).

Pearson’s chi-squared test showed a statistically significant difference between DIS and FC SNP haplotype frequencies (*χ*^2^ = 693.71, df = 10, *p* = 1.40 × 10^−142^; *p*-adjusted = 1.54 × 10^−141^) when DIS and FC haplotypes were each greater than or equal to five in frequency (Table 8). The results of this genetic association assay give qualitatively similar results to the genetic association assay performed on the same overlapping families using the edited 27-SNP haplotype method (see Section 3.4.1).

## 4. Discussion

The T1DGC MHC databases used in this study are two of the largest family-based dense SNP datasets available for allele and genetic evaluation. Furthermore, many of the genotyped pedigrees in these datasets include both parents and multiple children. These datasets thus provide a rich resource for direct observational pedigree-based haplotype phasing. The datasets have the added benefit of having a significant portion of the genotype data within a part of the human genome (the MHC) that is (a) highly gene-dense; (b) highly polymorphic; (c) the most completely characterized (on a population-based level) Mb region of the human genome; and, (d) linked to and/or associated with a wide variety of phenotypes-including the one (T1D) for which the datasets were designed.

Additionally, one of the two T1DGC datasets (the MHC Fine Mapping study) has HLA genotype data at 4-digit resolution, and there are many overlapping families with the other dataset (the ImmunoChip study). These facts, and prior knowledge of both the polymorphic nature and population-level polymorphic genomic architecture of the region, allowed us to correlate directly observed pedigree-phased edited SNP haplotypes with HLA haplotypes and longer-ranged CEHs. This provided a means of testing the degree to which the edited SNP haplotypes generated using our SNP editing process were representative of the same haplotypes defined by classical HLA-DR,DQ typing.

A major obstacle for pedigree-based observational definition of SNP haplotypes is the significant ambiguity in haplotype assignment, especially for biallelic SNPs, inherently created by relatively small pedigrees [28]. Furthermore, due to 1383 missing parents (“founders”), a large percentage of family genotype data was lacking in the ImmunoChip database we used. Nevertheless, genotype data can often be phased into defined haplotypes at many markers even using only haploidentical siblings. Using our strategy of prioritizing inclusion of high “coverage” SNPs (Supplementary Table S1; SNPs at which the percentage of fully-defined (-phased) haplotypes is maximal), we were able to define 58% of the haplotypes containing 27 SNPs.

Separately, we optimized the polymorphic nature of the resultant SNP haplotypes by choosing SNPs with a wide array of MAFs and removing any SNPs within any given MAF range that appeared to be “redundant.” Redundant SNPs are those that form only a biallelic SNP haplotype with any other SNP. That is, a SNP is redundant to another SNP if essentially all (≥95%) of the resultant independent fully-defined haplotypes contain only two of the theoretically four possible SNP haplotype combinations of the two tested SNPs. We maximized the final haplotype definition by removing the redundant SNP with the lower coverage.

Our results show that the method provides results remarkably similar in polymorphic detail as compared with classical four-digit HLA typing at three loci (*HLA*-*DRB1*, -*DQA1* and -*DQB1*) in the same genomic region. Indeed, the edited 27-SNP haplotypes could, for at least three separate HLA-DR,DQ haplotypes, distinguish pairs of different haplotype variants among identical 4-digit HLA-DR,DQ variants. For the pair of HLA-DR3,DQ2 variants (representing variants of the CEHs [HLA-B8,SC01,DR3] and [HLA-B18,F1C30,DR3]), this was not surprising. It was already known that these two DR3,DQ2 variants, while nearly identical in a 106 kb region overlapping with the 240 kb region we analyzed [27], have different alleles at another locus (*HLA*-*DRB3*) within the region we studied [13,14].

Conversely, the edited 27-SNP haplotypes could not distinguish variants of the HLA-DR4, DQ8 haplotype group (those that differ, at the third and fourth digits, in classical *HLA-DRB1* typing, but share the *HLA*-DQA1*03:01 and *HLA*-DQB1*03:02 alleles). This is also not particularly surprising. The T1DGC ImmunoChip dataset only included three SNPs within the *HLA*-*DRB1* locus itself. Although we used all three *HLA*-*DRB1* SNPs among our starting 37 SNPs (and triaged out one of them due to redundancy for the 27-SNP analysis), these SNPs were clearly insufficient to distinguish alleles that differ within a particular *HLA*-*DRB1* exonic sequence. The results suggest, however, that the DR4,DQ8 haplotypes may share a highly similar sequence throughout the HLA-DR,DQ region (other than at *HLA-DRB1*) in a way similar (although not within the same boundaries) to that of the DR3,DQ2 haplotype group [27].

Long-range (>1 Mb) human MHC haplotypes of highly fixed sequence markers existing at relatively high frequency among many geoethnic populations have been identified as CEHs [12,13,14,15,26]. Several MHC CEH pair variants and groups share sequence identity (e.g., Class III or HLA-DR,DQ blocks) surrounded both telomerically and centromerically by regions in which the CEHs differ significantly [12,13,14,26,27]. In this report, although we did not analyze all of the dominant CEHs in every edited SNP haplotype variant, our results clearly demonstrate that the SNP haplotype variants we evaluated in the 240 kb region are strongly genetically linked to and can act as surrogate markers of these long-range differences. As our results demonstrate (Table 5), the 27-SNP haplotypes in this single HLA class II region can be exploited to identify previously unreported putative CEHs.

T1D genetic association results, based on HLA-DR,DQ alleles and haplotype variants as well as individual SNP alleles and SNP haplotype variants, have been previously reported, in some cases using underlying data overlapping with those we analyzed. Our HLA-DR,DQ genetic association results (Table 8) among the 1067 families shared between the two T1DGC datasets largely parallel results from the two largest previously published analyses of T1D genetic association with HLA-DR,DQ haplotypes [3,25]. The earlier of the two publications was a 2007 meta-analysis of HLA-DR,DQ haplotype variant risk effects on T1D summarizing results from 38 studies conducted worldwide [3]. The relative distribution and ranks of T1D susceptibility haplotype variants as determined by DIS to FC ratios in our study essentially are identical to those found based on the 2007 study’s summary “patient to control” (P/C) ratios. Although we grouped all HLA-DR4,DQ8 haplotypes together, and there are a few specific HLA-DR4,DQ8 haplotypes in the 2007 meta-analysis that are not among the highest susceptibility group, the latter composed only 2% of the T1DGC dataset we used. The remaining 98% of HLA-DR4,DQ8 haplotypes we studied were all HLA-DR,DQ variants within the top 10 group of P/C ratios in the 2007 study [3].

In a 2008 report of HLA-DR,DQ haplotypes in a different subset of the T1DGC MHC Fine Mapping study [25], a family-based patient to control genetic association analysis showed a statistically different DR,DQ haplotype distribution among patients and controls in Caucasian (largely of European origin) subjects (*p* = 5 × 10^−124^) that parallels our study results. The study also found a rank hierarchy of haplotype risk for T1D (based on odds ratios) that was similar to both the 2007 meta-analysis [3] and the results we present here. In summary, the HLA-DR,DQ haplotype analysis we present here, with which we compare our edited 27-SNP haplotype analyses, is consistent with prior results.

The results of the analysis of HLA-DR,DQ haplotypes in overlapping families from the MHC Fine Mapping dataset (Table 8) were largely in parallel with the results of the analysis of the edited 27-SNP haplotypes (Table 7) in overlapping families from the ImmunoChip dataset. Thus, for both structural variant analysis and T1D genetic association analysis, the core method of edited SNP haplotypes provided a data source that was essentially as useful as 4-digit HLA-DR,DQ typing. Both methods of HLA class II variant designation captured variant 1 (DR4,DQ8) as the most frequent haplotype among all haplotypes and the most frequent DIS haplotype. Both methods also showed that variant 7 (DR15,DQ6) was one of the most protective haplotypes among all haplotypes and the most frequent haplotype among FC haplotypes. The genetic association assays based on HLA-DR,DQ haplotypes and edited 27-SNP haplotypes among overlapping families also gave qualitatively (and to a large extent, quantitatively) similar results. Variant 1 (among SNP haplotypes) and DR4,DQ8 (among HLA-DR,DQ haplotypes) both showed the highest ratio of DIS to FC haplotype frequency.

Finally, the edited 27-SNP haplotype variants among the overlapping families of the two T1DGC studies were representative of the edited 27-SNP haplotypes from the entire ImmunoChip dataset. Among the 34 most frequent fully-defined 27-SNP haplotype variants in the entire ImmunoChip dataset (Table 2), only 14 haplotype variants (3.9% of the total haplotypes) were not represented among SNP haplotypes in the overlapping families (Table 7). Among the 29 27-SNP haplotype variants existing at least once each as a DIS and as a FC haplotype in the entire ImmunoChip dataset (Table 6), only nine were not similarly represented among the 27-SNP haplotypes in the overlapping families (Table 7). Thus, the genetic association assays based on the edited 27-SNP haplotypes from the overlapping family subset showed qualitatively similar results to those edited 27-SNP haplotypes from the entire ImmunoChip dataset. Variants 1 and 7, both among overlapping families and in the entire ImmunoChip dataset, showed the largest differences in frequency ratios of DIS to FC haplotypes.

We did not compare our pedigree-phased and -defined structural haplotypes with those that might be generated from the same underlying genotype data using any of the numerous maximum likelihood statistical methods available to “impute” SNP haplotypes using unrelated individuals. However, for those investigators interested in testing or comparing the accuracy of various haplotype imputation methodologies (either with each other or with the directly phased and defined haplotypes produced herein), these two T1DGC datasets would seem to be ideal resources with which to conduct such studies. It would be a useful validation procedure for proponents of haplotype imputation, and any future designers of haplotype imputation methodologies, to use databases such as these two T1DGC MHC databases. Our prediction is that imputed haplotypes guessed at by maximum likelihood statistical methods using the same source genotypes used in this study would show quantitative inaccuracy as compared with the direct observational results presented here [15]. However, at the very least, such comparisons might lead to improved methodologies for haplotype imputation in those (unfortunately) common situations in which geneticists must use databases containing only genotype data from unrelated subjects.

In conclusion, we believe the method developed here to optimize SNP haplotype analysis may prove useful as a tool for a wide variety of end uses. The underlying method clearly provides structural information that parallels that of HLA typing and is, therefore, validated in the most intensively studied region of the human genome. The method can be used to analyze genetic association with a genetic phenotype, as we have presented here for the complex autoimmune disease T1D. The method can also be used to evaluate both regional and longer-range population-level genomic architecture. This opens up the entire human genome to the study of long-range AH/CEH structures that have thus far been limited almost entirely to the MHC.

## Figures and Tables

**Table 1:** T1DGC MHC Fine Mapping SNPs to distinguish B8,DR3 and B18,DR3 CEHs ^1^.

dbSNP Variants CEH	rs2076536	rs3117103	rs3135363	rs6901541	rs4999342	Cell Line Sequence		
B8,DR3	T	T	G	C	C	COX		
B18,DR3	C	A	A	T	T	QBL		
**T1DGC Variants** **CEH**	**rs2076536**	**rs3117103**	**rs3135363**	**rs6901541**	**rs4999342**	**% Dominant** **Sequence**	**% Other** **Sequences**	**% Unphased**
B8,DR3	1	1	3	2	2	95.0	0.8	4.2
B18,DR3	3	4	1	4	4	79.4	10.7	9.8

^1^ Seq = Sequence. Shown are the reference sequence (rs) SNP alleles for two different MHC conserved extended haplotypes (CEHs). dbSNP data were provided by NCBI (https://www.ncbi.nlm.nih.gov/snp/).

**Table 2:** The most frequent T1DGC ImmunoChip study edited 27-SNP haplotypes ^1^.

SNP Variant Name	Total (*n*)	Percentage	SNP Variant Name	Total (*n*)	Percentage
Variant 1	1786	28.3	Variant 19	45	0.7
Variant 2	1064	16.9	Variant 20	26	0.4
Variant 3	517	8.2	Variant 21	22	0.3
Variant 4	467	7.4	Variant 22	22	0.3
Variant 5	400	6.3	Variant 23	21	0.3
Variant 6	308	4.9	Variant 24	10	0.2
Variant 7	296	4.7	Variant 25	**8**	0.1
Variant 8	285	4.5	Variant 26	**8**	0.1
Variant 9	154	2.4	Variant 27	**8**	0.1
Variant 10	134	2.1	Variant 28	**8**	0.1
Variant 11	112	1.8	Variant 29	8	0.1
Variant 12	96	1.5	Variant 30	7	0.1
Variant 13	79	1.3	Variant 31	6	0.1
Variant 14	77	1.2	Variant 32	4	0.1
Variant 15	71	1.1	Variant 33	4	0.1
Variant 16	59	0.9	Variant 34	4	0.1
Variant 17	56	0.9	Variant 68	1	0.0
Variant 18	55	0.9			

^1^ These edited SNP haplotype variants are those that existed at *n* ≥ 4 in the entire T1DGC ImmunoChip study or were otherwise named in the main text (Variant 68). The SNP haplotype sequences for all of the edited SNP haplotypes named here are given in Supplementary Table S2.

**Table 3:** Major edited 27-SNP haplotypes shared by both T1DGC studies.

Edited SNP Haplo Rank	Variant Name	SNP Haplo Total (*n*)	% Defined SNP Haplos	Dominant HLA-DR,DQ Haplotype			
				*HLA-DRB1*	*HLA-DQA1*	*HLA-DQB1*	HLA Abbrev.
1	Variant 1	729	28.5	04:xx	03:01	03:02	DR4,DQ8
2	Variant 2	447	17.5	03:01	05:01	02:01	B8,DR3,DQ2
3	Variant 4	205	8.0	03:01	05:01	02:01	B18,DR3,DQ2
4	Variant 3	202	7.9	01:01	01:01	05:01	DR0101,DQ5
5	Variant 5	166	6.5	07:01	02:01	02:02	DR7,DQ2
6	Variant 6	124	4.8	04:xx	03:01	03:01/03:04	DR4,DQ7
7	Variant 7	116	4.5	15:01	01:02	06:02	DR15,DQ6
8	Variant 8	106	4.1	11:xx	05:01	03:01	DR11,DQ3
9	Variant 9	62	2.4	08:01	04:01	04:02	DR8,DQ4
10	Variant 10	53	2.1	13:02	01:02	06:04	DR1302,DQ6 var1
11	Variant 11	40	1.6	13:01	01:03	06:03	DR1301,DQ6 var1
11	Variant 12	40	1.6	16:01	01:02	05:02	DR16,DQ5
13	Variant 14	34	1.3	07:01	02:01	03:03	DR7,DQ3
14	Variant 13	30	1.2	13:01	01:03	06:03	DR1301,DQ6 var2
15	Variant 15	29	1.1	09:01	03:01	03:03	DR9,DQ3
16	Variant 17	23	0.9	12:01	05:01	03:01	DR12,DQ3
17	Variant 19	22	0.9	13:02	01:02	06:04	DR1302,DQ6 var2
18	Variant 18	21	0.8	14:01/14:04	01:01	05:03	DR14,DQ5
19	Variant 16	17	0.7	01:02	01:01	05:01	DR0102,DQ5
	TOTAL	2466	96.3				

**Table 4:** Major HLA-DR,DQ haplotypes shared in T1DGC studies: their dominant 27-SNP haplotype and their percentages ^1^.

DR,DQ Haplo Rank	HLA Haplo Abbrev.	DR,DQ Total (*n*)	% all DR,DQ Defined Haplos	Dominant SNP Haplotype	1st Total (*n*)	% of This DR,DQ Haplotype Group	% of Fully-Defined in This Group
1	DR4,DQ8	1024	27.4	Variant 1	722	70.5%	99.2%
2	All DR3,DQ2	950	25.4	Variant 2	441	46.4%	67.7%
3	DR0101,DQ5	290	7.8	Variant 3	185	63.8%	99.5%
4	DR7,DQ2	230	6.2	Variant 5	165	71.7%	100.0%
5	DR15,DQ6	188	5.0	Variant 7	110	58.5%	99.1%
6	DR11,DQ3	182	4.9	Variant 8	102	56.0%	98.1%
7	DR4,DQ7	155	4.1	Variant 6	106	68.4%	98.1%
8	DR1301,DQ6	108	2.9	Variant 11	40	37.0%	58.8%
9	DR1302,DQ6	104	2.8	Variant 10	51	49.0%	65.4%
10	DR8,DQ4	89	2.4	Variant 9	58	65.2%	90.6%
11	DR16,DQ5	62	1.7	Variant 12	40	64.5%	100.0%
11	DR7,DQ3	54	1.4	Variant 14	34	63.0%	94.4%
13	DR9,DQ3	45	1.2	Variant 15	29	64.4%	100.0%
14	DR14,DQ5	36	1.0	Variant 18	21	58.3%	87.5%
15	DR12,DQ3	30	0.8	Variant 17	22	73.3%	95.7%
16	DR0102,DQ5	27	0.7	Variant 16	17	63.0%	100.0%
	TOTAL	3574	95.7	TOTAL	2143		

^1^ The HLA haplotype abbreviations used here are those from Table 3 with minor exceptions. Here, the test haplotype is the HLA-DR,DQ (DR,DQ) haplotype. Therefore, for example, the entire DR3,DQ2 group is analyzed. The last column gives the percentage of each DR,DQ haplotype group represented by the dominant 27-SNP haplotype among all fully-defined 27-SNP haplotypes. The second most frequent 27-SNP haplotype and their percentages of each DR,DQ haplotype group as well as the total untyped or unphased 27-SNP haplotypes for each DR,DQ group are given in Supplementary Table S3.

**Table 5:** Dominant MHC CEHs in major 27-SNP edited haplotypes of the DR,DQ region ^1^.

SNP Haplo Var. Name	Dom. DR,DQ Haplo (*DRB1*,*DQA1*,*DQB1*)	SNP Haplo Total (*n*)	Dom. DR,DQ Haplo Total (*n*)	Dom. CEH of DR,DQ Var.	Dom. DR,DQ CEH Total (*n*; %)
Variant 1	*04:xx,03:01,03:02*	729	722	None	**
Variant 2	*03:01,05:01,02:01*	447	441	[HLA-C7,B8,SC01,DR3]	**
Variant 3	*01:01,01:01,05:01*	202	185	*	**
Variant 4	*03:01,05:01,02:01*	205	202	[HLA-C5,B18,F1C30,DR3]	**
Variant 5	*07:01,02:01,02:02*	166	165	*	**
Variant 6	*04:xx,03:01,03:01/03:04*	124	106	*	**
Variant 7	*15:01,01:02,06:02*	116	110	[HLA-C7,B7,SC31,DR15]	31; 52% ***
Variant 8	*11:xx,05:01,03:01*	106	102	None	**
Variant 9	*08:01,04:01,04:02*	62	58	[HLA-C7,B39,unk,DR8]	11; 19%
Var. 10	*13:02,01:02,06:04*	53	51	[HLA-C3,B40,SC02,DR13]	26; 51%
Var. 11	*13:01,01:03,06:03*	40	40	[HLA-C12,B38,SC21,DR13]	10; 25%
Var. 12	*16:01,01:02,05:02*	40	40	[HLA-C12,B39,unk,DR16]	9; 23%
Var. 13	*13:01,01:03,06:03*	30	27	[HLA-C3,B15,unk,DR13]	8; 30%
Var. 14	*07:01,02:01,03:03*	34	34	[HLA-C6,B57,SC61,DR7]	20; 59%
Var. 15	*09:01,03:01,03:03*	29	29	[HLA-C7,B7,unk,DR9]	5; 17%
Var. 16	*01:02,01:01,05:01*	17	17	[HLA-C8,B14,SC2(1,2),DR1]	11; 65%
Var. 17	*12:01,05:01,03:01*	23	22	[HLA-C5,B44,unk,DR12]	4; 18%
Var. 18	*14:01/14:04,01:01,05:03*	21	21	[HLA-C4,B35,unk,DR14]	6; 29%
Var. 19	*13:02,01:02,06:04*	22	22	[HLA-C7,B15,unk,DR13]	6; 27%
	TOTAL	2466	2394		

^1^ Dom. = Dominant; Haplo = Haplotype; Var. = Variant. * The dominant CEH of this group was not determined; ** The totals for these CEHs were not determined; *** Only 60 of 110 haplotypes were evaluated.

**Table 6:** Analysis of equalized fully-phased 27-SNP edited disease (DIS) and family control (FC) haplotypes from the ImmunoChip study ^1^.

SNP Haplo Var. Name	DIS Haplo (*n*)	FC Haplo (*n*)	Total (*n*)	DIS/FC Haplo Ratio	DIS Haplo Rank	FC Haplo Rank	χ^2^ *
Variant 1	916	238	1154	3.85	1	2	398.34
Variant 2	416	204	620	2.04	2	3	72.49
Variant 3	108	190	298	0.57	4	5	22.56
Variant 4	249	46	295	5.41	3	12	139.69
Variant 7	5	254	259	0.02	14	1	239.39
Variant 5	47	190	237	0.25	6	5	86.28
Variant 8	18	195	213	0.09	10	4	147.08
Variant 6	73	120	193	0.61	5	7	11.45
Variant 11	8	71	79	0.11	13	8	50.24
Variant 9	45	32	77	1.41	7	15	2.19
Variant 14	2	68	70	0.03	19	9	--
Variant 10	25	43	68	0.58	8	13	4.76
Variant 12	20	39	59	0.51	9	14	6.12
Variant 13	4	52	56	0.08	17	10	--
Variant 18	1	49	50	0.02	22	11	--
Variant 15	18	24	42	0.75	10	17	0.86
Variant 17	5	26	31	0.19	14	16	14.23
Variant 16	9	17	26	0.53	12	19	2.46
Variant 19	4	16	20	0.25	17	20	--
Variant 20	1	19	20	0.05	22	18	--
Variant 21	1	10	11	0.10	22	21	--
Variant 23	1	10	11	0.10	22	21	--
Variant 25	5	1	6	5.00	14	29	--
Variant 29	1	5	6	0.20	22	23	--
Variant 28	2	3	5	0.67	19	24	--
Variant 33	2	2	4	1.00	19	27	--
Variant 31	1	3	4	0.33	22	24	--
Variant 27	1	3	4	0.33	22	24	--
Variant 32	1	2	3	0.50	22	27	--
Misc. Haplos	15	72	87				
TOTAL	2004	2004	4008				1198.15

^1^ Misc. = Miscellaneous; Haplo = Haplotype; Var. = Variant. * Chi-squared statistic of DIS and FC SNP haplotypes each ≥ 5 in frequency.

**Table 7:** Analysis of equalized DIS and FC SNP haplotypes each ≥ 5 in frequency among overlapping families in both T1DGC studies ^1^.

SNP Haplo Var. Name	DIS Haplo (*n*)	FC Haplo (*n*)	Total (*n*)	DIS/FC Haplo Ratio	DIS Haplo Rank	FC Haplo Rank	χ^2^ *
Variant 1	373	102	475	3.66	1	2	154.61
Variant 2	169	84	253	2.01	2	3	28.56
Variant 4	105	24	129	4.38	3	9	50.86
Variant 3	44	72	116	0.61	4	5	6.76
Variant 7	1	103	104	0.01	17	1	--
Variant 5	21	78	99	0.27	6	4	32.82
Variant 8	11	70	81	0.16	8	6	42.98
Variant 6	26	47	73	0.55	5	7	6.04
Variant 9	17	16	33	1.06	7	13	0.03
Variant 11	2	30	32	0.07	13	8	--
Variant 10	10	16	26	0.63	9	13	1.38
Variant 12	6	18	24	0.33	10	12	6.00
Variant 13	2	20	22	0.10	13	10	--
Variant 18	1	19	20	0.05	17	11	--
Variant 15	3	12	15	0.25	12	15	--
Variant 17	1	11	12	0.09	17	16	--
Variant 19	4	5	9	0.80	11	18	--
Variant 16	2	6	8	0.33	13	17	--
Variant 28	2	1	3	2.00	13	19	--
Variant 27	1	1	2	1.00	17	19	--
Misc. Haplos	7	73	80				
TOTAL	808	808	1616				330.04

^1^ Misc.: Miscellaneous; Haplo: Haplotype; Var: Variant. * Chi-squared statistic of DIS and FC SNP haplotypes each ≥ 5 in frequency.

**Table 8:** Analysis of equalized DIS and FC HLA-DR,DQ haplotypes each ≥ 5 in frequency among overlapping families in both T1DGC studies ^1^.

DR,DQ Haplo Var. Name	DIS Haplo (*n*)	FC Haplo (*n*)	Total (*n*)	DIS/FC Haplo Ratio	DIS Haplo Rank	FC Haplo Rank	χ^2^ *
DR4,DQ8	508	87	595	5.84	1	6	297.88
DR3,DQ2	416	133	549	3.13	2	2	145.88
DR0405,DQ2	7	4	11	1.75	12	16	--
DR8,DQ4	30	26	56	1.15	5	12	0.29
DR13,DQ0604	21	30	51	0.70	7	10	1.59
DR0901,DQ0303	11	19	30	0.58	10	14	2.13
DR1,DQ0501	72	132	204	0.55	3	3	17.65
DR16,DQ0502	13	25	38	0.52	8	13	3.79
DR4,DQ7	27	68	95	0.40	6	8	17.69
DR7,DQ2	31	118	149	0.26	4	5	50.80
DR13,DQ0603	8	77	85	0.10	11	7	56.01
DR11,DQ0301	12	132	144	0.09	9	3	100.00
DR12,DQ0301	1	17	18	0.06	13	15	--
DR14,DQ0503	1	30	31	0.03	13	10	--
DR15,DQ0602	1	166	167	0.01	13	1	--
DR0701,DQ0303	0	49	49	0.00	16	9	--
Misc. Haplos	12	58	70				
TOTAL	1171	1171	2342				693.71

^1^ Misc.: Miscellaneous; Haplo: Haplotype; Var: Variant. *Chi-square statistic of DIS and FC HLA-DR,DQ haplotypes each ≥ 5 in frequency.

## Supplementary Materials

The following are available online at https://www.mdpi.com/2073-4409/8/8/835/s1. Table S1: SNPs used for edited haplotypes in HLA-DR/DQ region, Table S2: SNP sequences for the most frequent T1DGC ImmunoChip study edited 27-SNP haplotypes, Table S3: Major HLA-DR,DQ haplotypes shared in T1DGC studies: their secondary and unphased 27-SNP haplotypes and their percentages.
